# Data resource profile: Australian and New Zealand Hip Fracture Registry (ANZHFR)

**DOI:** 10.1177/11207000251370293

**Published:** 2025-10-31

**Authors:** Yushy Zhou, Christopher J Wall, Jamie Hallen, Rebecca Mitchell, Morag E Taylor, Lara A Harvey, Sarah Hurring, Michael C Wyatt, Nicola Ward, Stewart Fleming, Jacqueline C T Close

**Affiliations:** 1 Department of Orthopaedic Surgery, St. Vincent’s Hospital, Melbourne, Victoria, Australia; 2 Department of Surgery, The University of Melbourne, Melbourne, Victoria, Australia; 3 Australian and New Zealand Hip Fracture Registry (ANZHFR), Neuroscience Research Australia, Randwick, New South Wales, Australia; 4 Department of Orthopaedics, Darling Downs Health, Toowoomba, Queensland, Australia; 5 Rural Clinical School, University of Queensland, Toowoomba, Queensland, Australia; 6 Australian Institute of Health Innovation, Macquarie University, Sydney, New South Wales, Australia; 7 School of Health Sciences, Faculty of Medicine and Health, University of New South Wales, Sydney, New South Wales, Australia; 8 Neuroscience Research Australia, University of New South Wales, Sydney, New South Wales, Australia; 9 School of Clinical Medicine, Faculty of Medicine and Health, University of New South Wales, Sydney, New South Wales, Australia

**Keywords:** Australian and New Zealand Hip Fracture Registry (ANZHFR), cohort profile, data resource profile, hip fracture, neck of femur fracture, registry data

## Abstract

The Australian and New Zealand Hip Fracture Registry (ANZHFR) is a bi-national clinical quality registry established to enhance care and outcomes for older adults hospitalised with hip fractures. Since its inception in 2015, the ANZHFR has amassed data on over 120,000 hip fractures from 107 hospitals across Australia and New Zealand. This ongoing data collection adheres to an internationally agreed-upon minimum common dataset and incorporates indicators aligned with the Australian Commission on Safety and Quality in Health Care (ACSQHC) Hip Fracture Clinical Care Standard. These indicators reflect best-practice guidelines for hip fracture care. The registry tracks key processes and outcomes, including care at presentation, preoperative pain management, orthogeriatric involvement, timing of surgery, postoperative mobilisation, prevention of subsequent fractures, hospital discharge transitions, functional outcomes, quality of life at 120 days, and post-injury mortality. This manuscript serves as a foundational reference for all future publications using ANZHFR data, providing detailed insights into its structure, scope, and significance. Researchers and collaborators interested in utilising or contributing to the ANZHFR data are encouraged to contact the team at clinical@anzhfr.org.

## Introduction

Hip fracture is a leading cause of injury hospitalisation worldwide, contributing significantly to increased morbidity and mortality among older adults.^1^ In Australia, around 17,000 individuals experience a hip fracture each year, while in New Zealand, the figure is approximately 4000 annually.^2^ Recent studies indicate that people with a hip fracture are 3.5 times more likely to die within 12 months of the injury compared to similar individuals without a hip fracture.^3^ Additionally, a year after the fracture, 60% of people require assistance with basic activities of daily living such as feeding, dressing or toileting, and 80% need help with instrumental activities of daily living such as shopping, cooking or cleaning.^4,5^

Despite the substantial burden on both patients and healthcare systems, the management and care of hip fracture patients have historically lacked standardisation.^6^ This underscored the need for high-quality, standardised care, and led to the development of the *Blue Book on Fragility Fracture Care* – a joint initiative of the British Orthopaedic Association and British Geriatrics Society in 2007, which set out minimum care standards for fragility fracture care in the United Kingdom (UK).^7^ To facilitate effective benchmarking of these standards, the National Hip Fracture Database (NHFD) was launched the same year (2007) and is currently the largest hip fracture registry in the world.^8^ This was followed by the UK national guideline for hip fracture management, published in 2011 and subsequently updated in 2023.^9,10^

Following the success of the NHFD in improving patient care, key stakeholders involved in hip fracture care across Australia and New Zealand convened in 2011 with the aim of improving hip fracture care across the 2 nations.^11^ The agreed approach was to develop an NHMRC (National Health and Medical Research Council) endorsed hip fracture care guideline that could be used to inform a bi-national clinical care standard with a set of performance indicators that would be captured by a bi-national registry.

The NHMRC-endorsed guideline, published in 2014, was based on the existing UK guideline and adapted to suit the local context.^12^ The guideline informed the content of the Australian Commission on Safety and Quality in Health Care (ACSQHC) Hip Fracture Care Clinical Care Standard (the “Standard”) published in 2016.^13^ The Australian and New Zealand Hip Fracture Registry (ANZHFR or the “Registry”) was developed in parallel with the Standard and was initially funded through a grant supported by a competitive research grant from a private health insurer. Further funding was sought and obtained from state and national health departments and has continued to date.

As of 2024, all 22 eligible public hospitals in New Zealand and 78 of 91 (86%) eligible public hospitals in Australia are contributing data to the Registry. This widespread engagement highlights the growing commitment to using the ANZHFR as a tool for monitoring, auditing, and enhancing the quality of hip fracture care across Australia and New Zealand.

## Data sources and outcome measures

### ANZHFR data sources

The ANZHFR’s primary aim is to promote the delivery of high-quality, standardised hip fracture care through systematic data collection and analysis. To achieve this aim, the Registry gathers both patient-level and hospital-level data from participating hospitals across Australia and New Zealand. The focus is on collecting data pertaining to clinical care and reporting on care that represents the gold standard in hip fracture management, as determined by the Standard.

**Figure 1.**
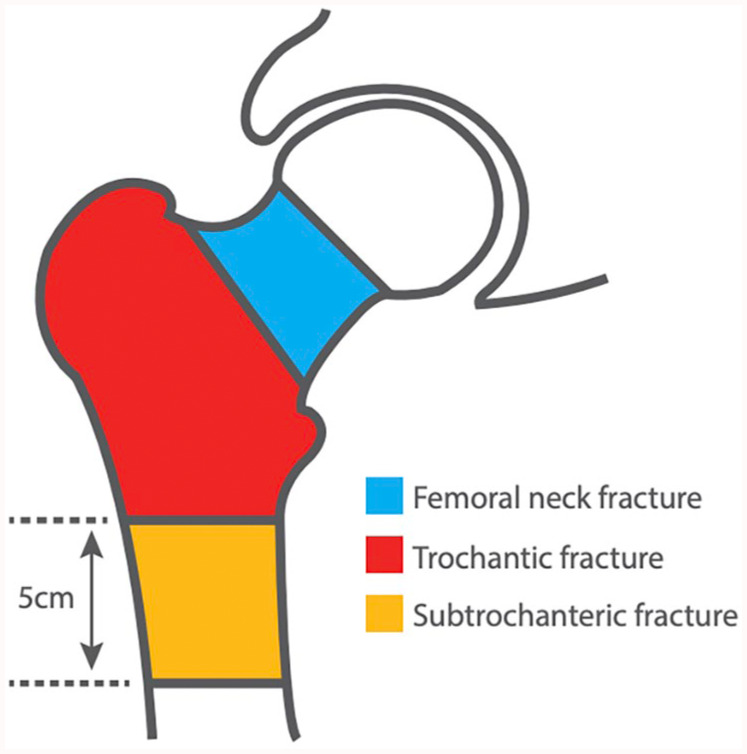
Zones of hip fracture as defined by the Australia and New Zealand Hip Fracture Registry.

The ANZHFR aims to collect data on every patient aged 50 years or older admitted to a participating hospital with a minimal trauma hip fracture, defined as a fracture resulting from trauma equivalent to or less severe than a fall from standing height. Patients may receive either surgical or non-surgical management for their fracture. The specific anatomical area used to define hip fracture is illustrated in Figure 1. Importantly, the ANZHFR collects data for the primary fracture of each hip, which means an individual can have two entries in the Registry – an entry for each hip.

### Minimum dataset

The ANZHFR developed a minimum data set (MDS) based on the existing NHFD dataset, the National Health Data Dictionary, and the Australasian Rehabilitation Outcomes Data Dictionary.^14,15^ This aimed to minimise the number of data variables collected but to simultaneously enable international benchmarking with other hip fracture registries, and to ensure that data of interest were classified using nationally-derived coding frames. The MDS has been developed to capture information relevant to the Standard and is comparable to the NHFD and other hip fracture registries across the world.^16,17^ Detailed data variable specifications, as well as data concordance documents describing changes made to data collection over time can be accessed on the ANZHFR website (publicly accessed at: https://anzhfr.org/data-access/).^18^

### Key outcome measures

The ANZHFR database is aligned with the quality indicators from the Standard and includes seven quality statements and a set of clinical indicators for providing safe and appropriate care to hip fracture patients. The quality statements relate to: (1) care at presentation; (2) preoperative pain management; (3) an orthogeriatric model of care; (4) timing of surgery; (5) postoperative mobilisation and weight-bearing; (6) minimising risk of another fracture; and (7) transition from hospital care. In general, the outcome measures are based on patient-level or hospital-level information.

The key patient-level information collected by the ANZHFR include:

- **Personal identifiers:** Information such as patient name, date of birth, ethnicity and medical record number to enable patient follow-up, data validation, and data linkage. *NB: Personal identifier data is not available for research purposes given the sensitivity of the information.*
- **Hospital information:** Details of the admitting hospital, including the type of ward the patient was admitted to and whether the hospital was public or private.
- **Timing of admission and surgery:** Data on the exact timing of patient admission and surgical intervention.
- **Pre-injury mobility and living circumstances:** Information on the patient's mobility status and living situation prior to the injury.
- **Preoperative and pre-admission cognition:** Assessments of the patient's cognitive status before admission and using a validated screening tool before surgery.
- **Operative details:** Comprehensive details of the surgical procedure, including the fracture pattern, type of surgery performed, the type of anaesthesia used, timing of surgery, and consultant surgeon presence. For non-operative patients the reason for non-operative management is recorded.
- **Postoperative care:** Data on the involvement of specialist geriatricians (also captured during pre-operative medical assessment), early mobility, delirium assessment, nutrition care, pressure injury, falls assessment, acute ward and hospital length of stay and discharge destination.
- **Postoperative outcomes:** Information on outcomes at 120 days after admission, such as mortality, walking ability, treatment for osteoporosis, re-operation and health-related quality of life (HRQoL). *NB: At time of publication, not all hospitals collect postoperative outcomes data.*

The ANZHFR captures a patient reported outcome measure (PROM) assessment using the EuroQol 5-Dimension 5-Level (EQ5D5L) at the 120-day follow-up.^19^ These scores offer valuable insights into patients' perceived health status, encompassing domains of mobility, self-care, usual activities, pain/discomfort, and anxiety/depression.

Hospital-level data is collected to understand how care of hip fracture patients occurs at a systems-level. The following hospital-level data points were collected between 2012 and 2023:

- **Hip fracture pathways:** The proportion of hospitals with hip fracture pathways.
- **Orthopaedic / geriatric shared care models:** A shared care structure between surgical and medical specialties.
- **Operating theatre availability:** Access to dedicated operating lists for hip fracture patients to minimise delay to surgery.
- **Weekend physiotherapy availability:** The proportion of hospitals with weekend access to physiotherapy services.
- **Patient information provision:** The proportion of hospitals that routinely provide written information on the treatment and after-care of patients with hip fractures.

Since 2024, the ANZHFR has streamlined its hospital-level data collection and now only collects data on three hospital-level questions. These questions relate to hip fracture pathways, pain pathways, and individualised care plans on discharge – all reportable quality indicators for the Standard. In addition, the ANZHFR conducts an annual linkage with the Australian National Death Index and New Zealand Ministry of Health to capture date and cause of death.

## Data processes and quality

### Data collection processes

The responsibility of data collection lies with each hospital. The primary data collection is carried out by clinicians caring for hip fracture patients. These are predominantly collected on paper-based forms and then entered by the participating hospital into the web-based Registry. Once the data has been inputted, the paper-based forms are securely destroyed. Data can also be directly entered into the online database or securely uploaded through an application processing interface.

The 120-day post-admission follow-up date is available via a follow-up calendar within the database. The 120-day follow-up consists of a phone call to the patient (or person responsible/residential aged care facility). The responses are entered into the ANZHFR online database by the team member who contacts the patient or their surrogate informant.

The ANZHFR operates on a waiver of consent (New South Wales, Queensland, South Australia, Tasmania, Northern Territory) and an opt-out consent (Western Australia, Victoria, New Zealand) process. Eligible participants are provided with a pamphlet regarding the ANZHFR that describes what type of data is collected, what the data will be used for (e.g. research) and how they can opt-out if they do not wish for their information to be included. There are a variety of methods for patients to opt-out, including telephone, email, or directly informing the health care team.

The implementation and maintenance of the ANZHFR are managed by a stakeholder group based at each participating hospital. This group typically comprises representatives from various disciplines, including medicine, surgery, nursing, allied health, hospital management, and other professionals involved in hip fracture care, such as anaesthetists, emergency physicians, and rehabilitation specialists. The Principal Investigator within this group takes overall responsibility for the implementation of the ANZHFR at each site. They are accountable for data governance, ensuring data is securely stored and de-identified before any local analysis. Each hospital only has access to data from their own institution for local analysis. The Principal Investigator also oversees regular data collection and submission, verifies the quality of the data provided to ANZHFR from their site and ensures that the data is used to deliver feedback aimed at improving the standard of care for hip fracture patients locally. Each hospital also appoints a Site Coordinator to support data collection and submission to ANZHFR.

### Data validation

Currently, the data validation process for the ANZHFR involves a combination of automated data checks, a yearly validation tool and an annual data quality audit. The automated data checks are designed to restrict incorrect data entry; for example, if a patient's date of birth indicates they are under 50 years old, a prompt will alert the user to reconsider the appropriateness or accuracy of the entry.

The annual data quality audit provides an additional layer of validation. Participation in the annual data quality audit is voluntary. This process includes several verification steps, such as temporarily removing approximately 10% of records and requesting the site to re-enter the data for those selected cases. The re-entered data is then compared with the original entries to assess the level of agreement between the 2 sets. A report is provided to the site detailing the consistency between the original and re-entered data. Once the comparison is completed, the temporarily removed records are restored. Additional steps include analysing the distribution of data to identify any outliers, cross-referencing with hospital or institutional records to detect suspicious data points, and, if necessary, re-contacting the patient or the responsible clinician to clarify questionable data entries.

### Data quality

In addition to data validation, which refers to the accuracy of the data being entered into the ANZHFR database, data completeness and data capture are also reviewed. For the ANZHFR, data completeness refers to the proportion of variables completed per patient record. In 2023, completeness was 99% for New Zealand hospitals and 97% for Australian hospitals. Data capture refers to the proportion of eligible patients captured by the ANZHFR. In New Zealand, data capture is cross-referenced with the MDS, with capture increasing from approximately 20% in 2016 to 89% in 2023. In Australia, data capture is difficult to measure due to jurisdictional differences between States and Territories. The ANZHFR aims to report this information in the future.

### Data evolution

The evolution of the ANZHFR dataset has been a continuous process to ensure its ongoing relevance and alignment with the changing landscape of hip fracture care. Initially, the MDS was based on the UK model. Any modifications to the dataset are systematically and carefully considered, with changes only occurring through an annual review process. These changes, if approved through the ANZHFR data management process, are implemented at 00:01 on the first day of the new year.

The reasons for updating variables are typically driven by: (1) updates to quality indicators as outlined in the revised Standard; (2) feedback from clinicians regarding issues with the wording or interpretation of existing variables; (3) the addition of new variables deemed critical for hip fracture care, such as the introduction of the Rockwood Clinical Frailty Scale;^20^ or (4) the removal of variables that no longer provide value. All changes are carefully documented, tracked, and archived, and can be accessed through the ANZHFR Data Forms for reference (publicly accessed at: https://anzhfr.org/data-forms/).^21^

## Data resource use

### Annual reports

Each year, the ANZHFR publishes an Annual Report which is publicly available on the Registry website (accessed at: https://anzhfr.org/registry-reports/).^22^ The Annual Report serves to update all stakeholders on the key findings and progress of hospitals participating in the ANZHFR for the year, including a comprehensive overview of the data collected to date. The Annual Report includes hospital identifiers if permission is granted by the hospital, (which in 2024 was 97% of all participating hospitals). In general, the Annual Report highlights advancements in quality of hip fracture care, areas of care that require improvement, and presents national and bi-national audit results. Additionally, the Annual Report details any changes in ANZHFR governance and outlines strategic directions and initiatives for the upcoming year.

### Sprint audits

The ANZHFR conducts an annual *Sprint Audit* focussing on a specific topic pertinent to hip fracture care where there is believed to be an opportunity to improve care. All hospitals except for Queensland hospitals (the administrative requirements of the Public Health Act prevent participation) are given the opportunity to participate. *Sprint Audits* are a tool to gather additional information, without the need to include data variables within the MDS. This “snapshot” or “sprint” approach enables a close-up view of one aspect of care in a short period of time. Data variables are temporarily added within a defined period to the routine registry data collection for eligible patients admitted to a participating hospital with a hip fracture. This enables data collection to inform hip fracture care improvements, whilst minimising burden on sites. Previous *Sprint Audits* have investigated nutrition, bone protection medication, acute rehabilitation, pre-operative fasting, and direct oral anticoagulant (DOAC) management. The results of the *Sprint Audits* are made publicly available on the ANZHFR website (accessed at: https://anzhfr.org/sprintaudits/).^23–27^

### Research

The ANZHFR has a strong research focus, collaborating with local and international researchers to produce high quality outputs focused on improving care for hip fracture patients. Clinical governance is provided by the ANZHFR Research Committee, formed by a multi-disciplinary team of academics and academic clinicians; and data and methodological governance is provided by the Data Management Committee, formed by a team of clinicians and academics with expertise in data science. To date, the Registry has produced over 30 publications, with a further 20 active projects underway and 7 projects under consideration. These key papers have identified the value of the Registry in improving the quality of hip fracture care, and reduced mortality associated with adherence to the Standard quality care indicators.^13,27^

#### Patient-reported experience measures pilot

As part of its commitment to improving patient outcomes, the ANZHFR has placed a strong emphasis on engaging with consumers to ensure their perspectives are integrated into quality improvement initiatives. The consumer arm, led by the ANZHFR Consumer Engagement Lead under the initiative *My Hip, My Voice*, actively represents consumer interests.^28^ Consumers are included in the ANZHFR governance structure to consult on outcomes that are important to them. For example, consumer input has been instrumental in the development of a consumer dashboard, ensuring that Registry data is accessible and understandable to all stakeholders (not just clinicians). Additionally, a consumer-focused video summarising the Annual Report is produced and released each year.^29^

## Strengths, limitations, and future directions

### Strengths

A key strength of the ANZHFR is its impact on improving care for hip fracture patients.^11^ The Registry has achieved 100% participation from hospitals in New Zealand and over 85% participation from Australian public hospitals, and this high level of engagement ensures that the data collected is comprehensive and representative. The Registry’s alignment with the Standard allows participating sites to consistently measure and benchmark their performance using the Annual Reports, with access to real-time local data via dashboards to foster ongoing quality improvement activities. The Registry has demonstrated its ability to enhance care quality, as evidenced by improvements in areas such as preoperative cognitive and delirium assessments, the use of nerve blocks, and bone protection medication upon discharge.^13^

Another key strength of the ANZHFR is its support from major stakeholders (e.g. ACSQHC and the participating hospitals) and the involvement of consumers, ensuring broad-based engagement and accountability. The Registry's transparent approach, with almost all hospitals named and their data publicly available, enhances trust and encourages improvements across the board. Additionally, the ANZHFR allows for international benchmarking through standardised data variables, providing a global context for performance evaluation.

### Limitations

While highly successful in many areas, the ANZHFR has several limitations that must also be acknowledged. 1 notable limitation is the limited engagement of private hospitals in Australia, alongside the ongoing efforts to achieve full participation from Australian public hospitals.

Missing data remains a challenge and addressing this issue is critical for providing a complete picture of hip fracture care.^30^ Part of this issue stems from the voluntary nature of PROMs and 120-day follow-up data collection. Additionally, while sites have access to a real-time dashboard for performance data, some hospitals are not actively using this as a tool to drive quality improvement initiatives, limiting the full potential of this resource.

Another limitation is the exclusion of patients under 50 years old from data capture. This can lead to missed opportunities for tracking outcomes in younger patients who experience hip fractures due to frailty from conditions such as younger onset dementia or neuromuscular diseases.^31^ Though the principles of care are similar, the lack of specific data for this subgroup may hinder understanding of their outcomes.

### Future directions

Looking ahead, the ANZHFR has several key areas of focus for future growth and development. One important direction is the increased engagement of consumers, which will strengthen the patient-centred approach and ensure that the voices of those affected by hip fractures are reflected in care improvements. Another priority is the linkage of registry data with administrative datasets, such as hospital admissions, residential aged care, and the Pharmaceutical Benefits Scheme (PBS) in Australia, which will provide a more comprehensive understanding of hip fracture care and recovery, including long-term outcomes and medication use.

Collaboration with international registries, such as through the OMOP (Observational Medical Outcomes Partnership) database, will further enhance the ability to benchmark performance on a global scale and share best practices across borders.^32^ This provides an open community data-sharing model with standardised data structures and content. Additionally, efforts to integrate the Registry with electronic medical records will streamline data collection by enabling automated data uploads.^33^ This integration will improve the timeliness and accuracy of data, making it easier for hospitals to contribute to and use the Registry for real-time quality improvement efforts.

## Data access

### Research data access

Access to data from the ANZHFR is governed by the ANZHFR Data Access Policy.^18^ Before applying, applicants must contact the ANZHFR Registry Manager to discuss their research question and review the list of already approved and pending research projects. Local Human Research Ethics Committee (HREC) approval must be granted prior to ANZHFR data access. New projects are reviewed by both the ANZHFR Data Committee and Steering Committee prior to approval.

The Data Access Policy ensures privacy and confidentiality by requiring that data access requests comply with HREC approvals and Research Governance Office requirements. Protecting participant privacy is a priority for the Registry. Applicants must ensure secure data storage, include a data disposal date in their requests, and avoid attempts to re-identify any individuals. Research proposals must provide ethics approval, a schedule for reporting results, and submit the HREC annual progress report to the ANZHFR throughout the duration of the project. Interested researchers and collaborators can complete an ANZHFR Data Policy and Access Request form, accessed via the Registry manager or email clinical@anzhfr.org.
